# Nipah virus infection and glycoprotein targeting in endothelial cells

**DOI:** 10.1186/1743-422X-7-305

**Published:** 2010-11-08

**Authors:** Stephanie Erbar, Andrea Maisner

**Affiliations:** 1 Institute of Virology, Philipps University of Marburg, Germany

## Abstract

**Background:**

The highly pathogenic Nipah virus (NiV) causes fatal respiratory and brain infections in animals and humans. The major hallmark of the infection is a systemic endothelial infection, predominantly in the CNS. Infection of brain endothelial cells allows the virus to overcome the blood-brain-barrier (BBB) and to subsequently infect the brain parenchyma. However, the mechanisms of NiV replication in endothelial cells are poorly elucidated. We have shown recently that the bipolar or basolateral expression of the NiV surface glycoproteins F and G in polarized epithelial cell layers is involved in lateral virus spread via cell-to-cell fusion and that correct sorting depends on tyrosine-dependent targeting signals in the cytoplasmic tails of the glycoproteins. Since endothelial cells share many characteristics with epithelial cells in terms of polarization and protein sorting, we wanted to elucidate the role of the NiV glycoprotein targeting signals in endothelial cells.

**Results:**

As observed *in vivo*, NiV infection of endothelial cells induced syncytia formation. The further finding that infection increased the transendothelial permeability supports the idea of spread of infection via cell-to-cell fusion and endothelial cell damage as a mechanism to overcome the BBB. We then revealed that both glycoproteins are expressed at lateral cell junctions (bipolar), not only in NiV-infected primary endothelial cells but also upon stable expression in immortalized endothelial cells. Interestingly, mutation of tyrosines 525 and 542/543 in the cytoplasmic tail of the F protein led to an apical redistribution of the protein in endothelial cells whereas tyrosine mutations in the G protein had no effect at all. This fully contrasts the previous results in epithelial cells where tyrosine 525 in the F, and tyrosines 28/29 in the G protein were required for correct targeting.

**Conclusion:**

We conclude that the NiV glycoprotein distribution is responsible for lateral virus spread in both, epithelial and endothelial cell monolayers. However, the prerequisites for correct protein targeting differ markedly in the two polarized cell types.

## Background

NiV is a biosafety-level 4 (BSL-4) categorized zoonotic paramyxovirus that first appeared in 1998 in Malaysia. During this outbreak, NiV was transmitted from its natural reservoir, fruit bats, to pigs which developed acute neurological and respiratory syndromes [1]. The human outbreak followed the contact with infected pigs and resulted in febrile encephalitic illnesses with high mortality rates [2]. In more recent NiV outbreaks in India and Bangladesh, the virus was directly transmitted from pteropoid bats to humans [3].

NiV enters the body via the respiratory tract, then overcomes the epithelial barrier and spreads systemically. Whereas epithelial cells are important targets in primary infection, and replication in epithelial surfaces of the respiratory or urinary tract is essential in late phases of infection for virus shedding and transmission, endothelial cells represent the major target cells during the systemic phase of infection which is characterized by a systemic vasculitis and discrete, plaque-like, parenchymal necrosis and inflammation in most organs, particularly in the central nervous system (CNS). The pathogenesis of NiV infection appears to be primarily due to endothelial damage, multinucleated syncytia and vasculitis-induced thrombosis, ischaemia and microinfarction in the CNS, allowing the virus to overcome the blood-brain-barrier (BBB) and to subsequently infect neurons and glia cells in the brain parenchyma [4,5].

A major characteristic of epithelial and endothelial target cells is their polarized nature. Epithelial as well as endothelial cells have structurally and functionally discrete apical and basolateral plasma membrane domains. To maintain the distinct protein compositions of these domains newly synthesized membrane proteins must be sorted to the sites of their ultimate function and residence [6]. Also viral proteins can be selectively expressed at either apical or basolateral cell surfaces thereby restricting virus budding or cell-to-cell fusion with significant implications for virus spread and thus for pathogenesis.

As most paramyxoviruses, NiV encodes for two envelope glycoproteins: The glycoprotein G is required for binding to the cellular NiV receptors ephrin-B2 and -B3 [7-10]. The fusion protein F is responsible for pH-independent fusion processes during virus entry and virus spread via cell-to-cell fusion. To become fusion active, the F protein precursor must be proteolytically activated by host cell cathepsins within endosomes. F cleavage thus depends on a functional tyrosine-based endocytosis signal in the F cytoplasmic tail (Y_525_RSL; [11-15]).

Interestingly, the same motif is also involved in basolateral sorting of the F protein in polarized epithelial cells. In a very recent study in which we attempted to elucidate the mechanisms of NiV spread from and within polarized epithelia, we demonstrate that infection of polarized cells induces foci formation with both glycoproteins located at lateral membranes of infected cells adjacent to uninfected cells. This suggested a direct spread of infection via lateral cell-to-cell fusion. Supporting this model, we could identify basolateral targeting signals in the cytoplasmic domains of both NiV glycoproteins: In the G protein, we identified a cytoplasmic di-tyrosine motif at position 28/29 which mediates polarized targeting. In the F protein, as mentioned above, tyrosine 525 within the endocytosis signal is responsible for basolateral sorting.

Since endothelial cells have a polarized phenotype comparable to epithelial cells, and endothelial infection in the CNS is mostly responsible for the pathogenesis of the NiV infection *in vivo*, we wanted to analyze the spread of NiV in endothelia and to evaluate the role of the tyrosine-based signals recently identified to be important for NiV glycoprotein targeting and cell-to-cell spread in polarized epithelial cells.

## Results

### NiV infection of polarized endothelial cells causes syncytia formation and increases transendothelial permeability

**Figure 1:**
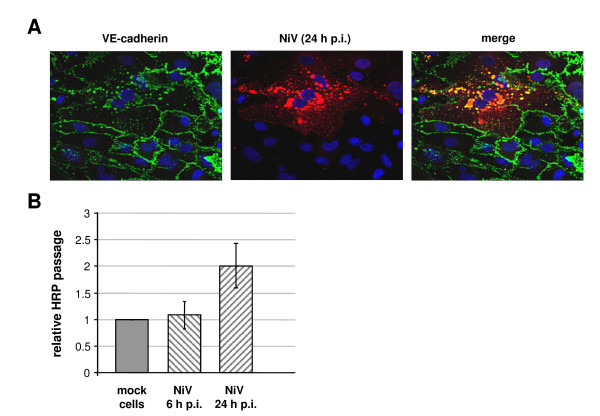
**NiV infection and permeability of primary endothelial cells**. Primary porcine brain microvascular endothelial cells (PBMEC) were cultured on fibronectin-coated filter supports for 6 days. Then, cells were infected with NiV at a m.o.i. of 0.5. (A) At 24 h p.i., cells were fixed with 4% PFA for 48 h. Subsequently, cells were stained with an NiV-specific guinea pig antiserum and AlexaFluor 568-conjugated secondary antibodies. After permeabilization with 0.1% TX-100, cell junctions were visualized with a monoclonal antibody directed against VE-cadherin and AlexaFluor 488-conjugated secondary antibodies. Magnification, 400×. (B) Effect of NiV infection on the permeability of endothelial monolayers. HRP (5 μg/ml) was added to the apical filter chamber of a filter insert with uninfected PBMEC (mock cells), or to filter inserts with NiV-infected PBMEC at 6 or 24 h p.i. (NiV 6 h p.i. or NiV 24 h p.i.). Apical-to-basolateral HRP passage was quantified by measurement of the HRP activity in the medium of the basal filter chamber every 10 min, and is given as means of 3 independent experiments normalized to the HRP concentration in mock-infected control wells.

Primary brain capillary endothelial cells have the closest resemblance to brain endothelia *in vivo *and exhibit excellent characteristics of the BBB at early passages. We therefore performed our initial studies in primary brain microvascular endothelial cells (PBMEC) freshly isolated from pig brains. Non-passaged PBMEC were cultivated on fibronectin-coated transwell filter supports with a pore size of 1 μm until full confluency and polarization were reached (6 days). Then, cells were infected with NiV at a multiplicity of infection (m.o.i.) of 0.5 under BSL-4 conditions. At 24 h p.i., the samples were inactivated with 4% PFA for 48 h. Virus-positive cells were immunostained with a NiV-specific polyclonal guinea pig antiserum and AlexaFluor 568-conjugated secondary antibodies. To visualize cell junctions, cells were permeabilized and VE-cadherin was co-stained with a specific monoclonal antibody and an AlexaFluor 488-conjugated secondary antibody. In agreement with the *in vivo *studies in NiV-infected pigs [16,17], NiV infection caused a foci formation in the cultured primary porcine brain endothelia (Figure 1A). As observed previously in epithelial cells [18], cell junction staining was lost within the NiV-positive foci indicating a virus-induced cell-to-cell fusion (syncytia formation). Because brain microvascular endothelial cells as a major component of the BBB develop complete intercellular tight junction complexes, have no fenestrations, and are scarce of transcytotic vesicles [19,20], entry of most molecules from blood to brain parenchyma is impeded. To investigate the effect of NiV infection on the transendothelial permeability, we used a peroxidase (HRP) leak assay [21]. PBMEC were seeded on filter supports and were infected with NiV. At 6 h and 24 h p.i., the culture medium in the apical filter chamber was replaced by medium containing 5 μg HRP per ml. Apical-to-basolateral HRP passage through the endothelial monolayer was monitored over the time and is given as the relative HRP passage normalized to the HRP passage through mock-infected cells. As shown in Figure 1B, we did not observe a significant increase in HRP permeability in PBMEC infected for 6 h, a time point of infection at which virus replication is already ongoing but newly synthesized viral proteins and syncytia formation were not yet detectable (data not shown). In contrast, at 24 h p.i., when syncytia formation and the accompanying cytopathic effect were clearly detectable (Figure 1A), we found an about 2-fold increase in transendothelial permeability (Figure 1B; NiV 24 h p.i.). These findings indicate that NiV infection does not drastically influence endothelial permeability and barrier functions at early time points of infection. Only after productive replication and pronounced syncytia formation interfering with cell monolayer integrity, transendothelial permeability is increased.

### Bipolar expression of the viral glycoproteins in primary and immortalized NiV-infected endothelial cells

**Figure 2:**
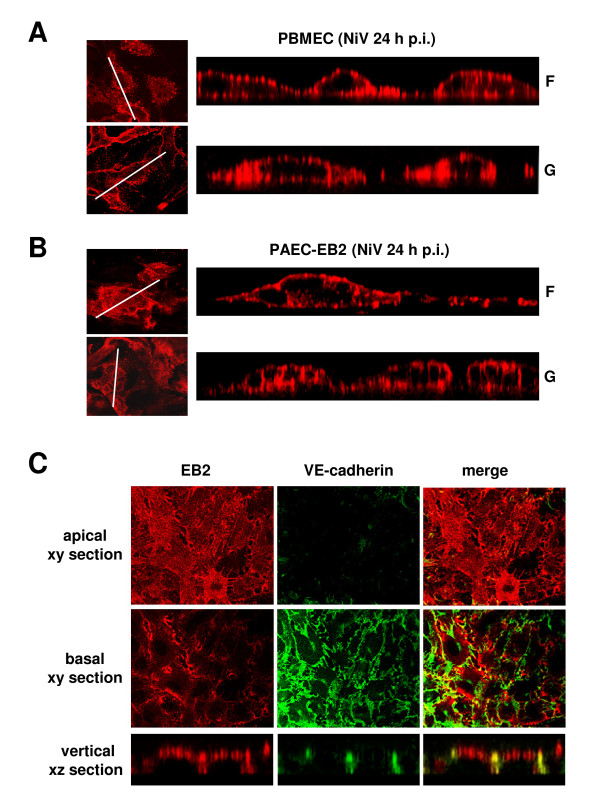
**Distribution of the NiV glycoproteins and the NiV receptor EB2 on the surface of polarized endothelial cells**. PBMEC (A) and PAEC-EB2 (B and C) were cultured on filter supports for 6 or 5 days, respectively. (A, B) Polarized cell cultures were infected with NiV at a m.o.i. of 0.5. At 24 h p.i., cells were inactivated and fixed with 4% PFA and then incubated from both sides with monoclonal antibodies directed either against the F or the G protein, followed by incubation with AlexaFluor 568-conjugated secondary antibodies. Confocal horizontal (xy) sections through the apical part of the cell monolayer are shown in the left panel. White lines indicate the area along which vertical sections were recorded. Vertical (xz) sections through the foci are shown on the left panel. (C) Cells were fixed and surface-stained from both sides with a EB2-specific ligand (EphB4/Fc) and a AlexaFluor 568-labelled secondary antibody. Then cells were permeabilized and incubated with a VE-cadherin specific antibody and a AlexaFluor 488-conjugated secondary antibody. Confocal horizontal (xy) and vertical (xz) sections are shown.

The finding that NiV infection rapidly leads to syncytia formation in endothelial cells suggests a lateral virus spread via cell-to-cell fusion due to (baso)lateral expression of F and G. To determine the surface distribution of the glycoproteins, NiV-infected PBMEC were fixed with 4% PFA and probed from the apical and basolateral side with a specific monoclonal antibody against either the F or the G protein, and AlexaFluor 568-conjugated secondary antibodies. Confocal horizontal sections through the apical part of NiV-positive foci and vertical sections for the F and G protein staining are shown in Figure 2A and 2B. The side views in the right panels clearly demonstrate a bipolar distribution of both NiV glycoproteins on the surface of infected PBMEC. Since cell-to-cell fusion requires the presence of both viral glycoproteins at contacting or lateral membranes this explains the observed syncytia formation. To evaluate if NiV-induced syncytia formation and bipolar glycoprotein expression is restricted to brain or microvascular endothelia, or is also observed in other endothelial cells, we infected immortalized porcine aortic endothelial cells stably expressing the NiV receptor ephrin-B2 (PAEC-EB2 [22,23]). As in PBMEC, NiV F and G proteins were expressed in a bipolar fashion and caused a pronounced syncytia formation (Figure 2B). Since virus-induced cell-to-cell fusion in polarized cell monolayers is only possible if viral receptors are expressed at lateral cell sides, we analyzed the distribution of the major NiV receptor EB2. In agreement with this hypothesis, the NiV receptor was found to be localized on the apical cell sides and at interendothelial cell junctions, partly colocalizing with VE-cadherin (Figure 2C).

### Distribution of NiV wildtype and mutant F and G proteins in polarized endothelial cells upon single expression differs from the distribution recently described in epithelia

**Figure 3:**
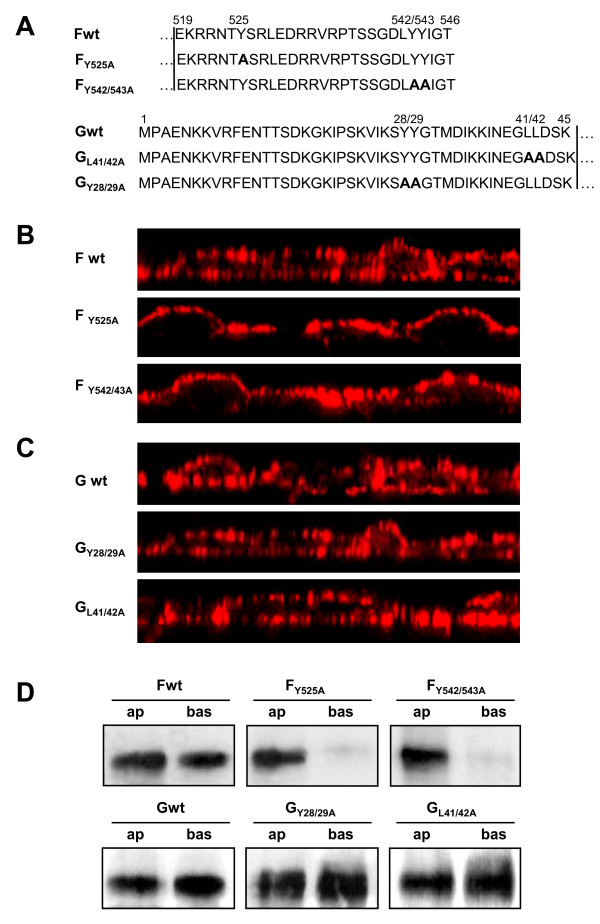
**Surface distribution of wild-type and mutant F and G proteins**. (A) Amino acid sequences of the cytoplasmic domains of wild-type and mutant F and G proteins. Numbers above the sequences indicate amino acid positions. Boldface letters indicate exchanged amino acid residues. Vertical lines indicate the beginning of the predicted transmembrane domains. (B and C) Surface distribution of wild-type F and G proteins in polarized endothelial cells. PAEC stably expressing either wild-type or mutant NiV F (B) or G (C) were grown on filter supports for 5 days and then incubated with a NiV-specific antiserum from the apical and basolateral sides without prior fixation. Surface-bound antibodies were detected with AlexaFluor 568-conjugated secondary antibodies. Confocal vertical sections through the cell monolayers are shown. (D) Cell surface proteins were labelled with S-NHS biotin from either the apical (ap) or the basolateral (bas) side. After cell lysis, F and G proteins were immunoprecipitated with NiV-specific antibodies. Precipitates were analyzed by SDS-PAGE under reducing conditions, transferred to nitrocellulose, and probed with peroxidase-conjugated streptavidin and chemiluminescence.

Previous studies in polarized epithelial cells had shown that bipolar distribution of the NiV glycoproteins in infected epithelia is correlated with a predominant basolateral expression of the F and G proteins in the absence of virus infection ([18]; table 1). Upon single expression of the glycoproteins, basolateral sorting was shown to depend on cytoplasmic tyrosine-based targeting motifs: Y_525 _in the F protein and di-tyrosine Y_28/29 _in the G protein. Mutations in the two other potential basolateral sorting motifs, a di-tyrosine motif in the F protein (Y_542/543_) and a di-leucine motif in the G protein (L_41/42_) had no influence on basolateral sorting (table 1). Epithelial and endothelial cell types share common characteristics since they both form junctional complexes that seal off an apical surface area and both cell types support a vectorial exchange of substances between apical and basolateral compartments. However, sorting of membrane proteins not always follows the same rules. Several cellular proteins, such as the transferrin receptor, the polymeric immunoglobulin receptor and tissue factor, which are selectively expressed on the basolateral surface of epithelial cells are oppositely targeted to the apical membrane of endothelial cells [24-26]. It thus remains to be elucidated if the cytoplasmic tyrosine residues in the NiV glycoproteins, shown to act as basolateral sorting signals in epithelial cells, have the same function in endothelial cells. We therefore decided to analyze the sorting of F and G proteins with mutated potential tyrosine and leucine-dependent sorting signals in polarized endothelial cells. The cytoplasmic tail sequences of wildtype and mutant proteins are depicted in Figure 3A. Since transient expression in primary endothelial cells is extremely inefficient and often interferes with cell polarization, we generated PAEC clones stably expressing either wildtype or mutant NiV glycoproteins. To monitor the targeting of the expressed proteins, the cells were cultured on filter supports. At 5 days after seeding, the cells had formed confluent and polarized monolayers and were labeled without prior fixation with NiV-specific antibodies and AlexaFluor 568-conjugated secondary antibodies from both, the apical and basolateral side. Confocal vertical sections through the cell monolayers are shown in Figure 3B and 3C. As in the infection (Figure 2), wildtype F was expressed bipolar upon single expression (Figure 3B; Fwt). Interestingly, mutations in both Y-based signals in the F protein (Y_525 _and YY_542/543_) led to an apical F redistribution (Figure 3B; F_Y525A_; F_Y542/543A_). This contrasts with our recent findings in polarized epithelial cells which showed that polarized distribution of the NiV F protein only depends on Y_525 _but not on the di-tyrosine motif at position 542/543 ([18]; table 1). Also, the distribution of the G protein is differently affected by the cytoplasmic tail mutations. Mutant G_Y28/29A _that was previously found to be sorted apically in polarized epithelial cells showed bipolar expression in PAEC as did the wildtype G protein (Figure 3C; Gwt; G_Y28/29A_). Mutation in the di-leucine motif did also not affect the bipolar G distribution (Figure 3C; G_L41/42A_).

**Table 1:** Summary and comparison of NiV infection and glycoprotein targeting in polarized epithelial and endothelial cells.

	Epithelial cells (Weise et al., 2010)	Endothelial cells (this study)
Foci formation in NiV-infected polarized cell monolayers	yes	yes

Glycoprotein distribution in NiV-infected polarized cells		
F protein	bipolar	bipolar
G protein	bipolar	bipolar

Glycoprotein distribution in polarized cells upon single expression		
F protein	basolateral	bipolar
G protein	basolateral	bipolar

Distribution of glycoproteins with mutations in potential cytoplasmic sorting signals		
F_Y525A_	apical	apical
F_Y542/543A_	basolateral	apical
G_Y28/29A_	apical	bipolar
G_L41/42A_	basolateral	bipolar

To confirm the distribution of the F and G proteins by a different method, we performed a selective surface biotinylation. For this, PAEC clones were cultured on filter supports and labeled from either the apical or basolateral side with non-membrane-permeating biotin. After cell lysis and immunoprecipitation, F and G proteins were separated by SDS-PAGE and blotted to nitrocellulose membranes. Surface-biotinylated glycoproteins were then detected with peroxidase-conjugated streptavidin. As shown in Figure 3D, similar amounts of biotinylated F wildtype protein could be detected on both surfaces (53.8% apical and 46.2% basolateral). Confirming the results obtained by confocal microscopy, both F mutants were predominantly detected after apical surface biotinylation (>95%). Also in agreement with the confocal immunofluorescence analysis, distribution of the wildtype and both mutant G proteins was bipolar, with slightly more of the G proteins expressed on the basolateral surfaces (60-65%).

## Discussion

In agreement with our previous findings in polarized epithelial cells, this study provides evidence that bipolar targeting of the two NiV surface glycoproteins is responsible for lateral spread of infection and syncytia formation in polarized endothelial cell monolayers. Interestingly, mutations in potential cytoplasmic sorting signals differently affected F and G targeting in endothelial cells compared with epithelial cells. Exchange of both tyrosine signals in the F protein led to an apical redistribution in endothelial cells whereas only tyrosine 525 is involved in targeting in epithelial cells. Neither the di-tyrosine nor the di-leucine motif in the cytoplasmic tail of the G protein influenced G distribution in endothelial cells while the di-tyrosine motif is essential for (baso)lateral expression in polarized epithelia (summarized in table 1).

The most unique diagnostic finding during a NiV infection is the presence of multinucleated endothelial cells in several organs. This widespread vasculitis, as key event in the pathogenesis of NiV infection, seems to be a consequence of infection of the vascular endothelial and smooth muscle cells [5,17]. NiV infection in the CNS is characterized by vasculitic vessels, numerous infected neurons and necrotic plaques suggesting that viral spread in brain endothelia is responsible for the disruption of the BBB, thus for virus dissemination into the brain parenchyma. The observed NiV-induced endothelial damage by foci or syncytia formation in cultured PBMEC which is accompanied by an increase in the transendothelial permeability late in infection is in agreement with the observed break in the BBB as well as the infiltration of leukocytes in small brain vessels during NiV infection *in vivo *[17,27]. In contrast to the endothelial damage and loss of barrier function caused by hemorrhagic viruses such as Marburg or Ebola viruses, TNF-α secretion from virus-infected macrophages appeared not to be required [21]. Among other paramyxoviruses also invading the CNS [11,28-30], at least the entry of measles virus into the CNS is also thought to be facilitated by direct infection and damage of brain endothelia [31,32].

NiV spread of infection across the lateral junctions of endothelial cells via cell-to-cell fusion was found to be as efficient as in epithelial cells and is, as in epithelia, due to a bipolar F and G expression. However, the targeting information required for functional glycoprotein expression at interendothelial cell contact sides appeared to be different from the tyrosine-dependent targeting signals required for basolateral or bipolar expression and cell-to-cell fusion activity in polarized epithelial cells (table 1). Whereas basolateral targeting of the F protein in polarized epithelial cells only depends on the Y_525 _which is also involved in the clathrin-mediated endocytosis of the F protein, and is thus essential for proteolytic activation by endosomal cathepsins [12,15,18], bipolar expression in endothelia further requires the tyrosines at positions 542/543. In contrast, the di-tyrosine motif in the G protein which we found to be important for basolateral G expression in epithelial cells is not required for bipolar expression of G in endothelia. Our findings that the Y-based sorting signals in the cytoplasmic tails of F and G do not play the same roles in epithelial and endothelial cells thus support the reports on cellular proteins describing that polarized transport and also recognition of protein sorting signals are not necessarily the same in epithelial and endothelial cells and can thus not be predicted in advance [26,33].

Since cell-to-cell fusion depends on the functional expression of both NiV glycoproteins at lateral contact sides between polarized cells, apical retargeting of just one glycoprotein is sufficient to prevent fusion and syncytia formation in polarized monolayers. Consequently, mutations in the viral glycoproteins that differently affect sorting also affect the fusogenic properties in the two polarized cell types.

## Conclusion

Spread of NiV infection within the two most important target cell types of the *in vivo *infection, endothelial and epithelial cells, occurs via cell-to-cell fusion, and is mediated by NiV glycoproteins expressed a the cell-cell contact sides. Nevertheless, sequence requirements for the targeting of the NiV glycoproteins is different supporting the idea that despite the polarized phenotype of epithelial and endothelial cells, protein targeting information required for correct sorting differs and cannot simply be predicted.

## Methods

### Cell culture and virus infection

PBMEC (primary porcine microvascular endothelial cells), freshly isolated from pig brain according to the protocol described by Bowman et al. [34] were cultured in Medium 199 (Gibco) supplemented with 20% FCS, 2 mM L-glutamine, 100 U penicillin ml^-1 ^and 100 mg streptomycin ml^-1 ^(all materials from GIBCO). PAEC (porcine aortic endothelial cells) were cultured in DMEM/F12 + GLUTAMAX (GIBCO) supplemented with 10% FCS, penicillin and streptomycin.

For polarized growth of endothelial cells, 0.4 or 1 μm pore size filter supports (ThinCerts™ Tissue Culture Inserts; Greiner Bio-One) were coated with 20 μg fibronectin per ml for 45 min at RT and for 16 h at 4°C. After extensive washes with PBS, cells were seeded on the filter supports and cultured at 37°C.

The NiV strain used in this study was isolated from human brain (kindly provided by J. Cardosa) and propagated as described previously [35]. For NiV infection studies, PBMEC and PAEC were grown on filter supports for 6 or 5 d, respectively: Medium was exchanged daily until they had developed a fully polarized phenotype. Cells were then infected with NiV by adding a multiplicity of infection (m.o.i.) of 0.5 to the apical filter chamber for 1.5 h at 37°C. Unbound virus was removed by extensive washings and cells were cultured with DMEM containing 2% FCS at 37°C. All work with live NiV was performed under biosafety-level 4 (BSL4) conditions.

### Permeability assay

PBMEC were seeded on the fibronectin-coated 1 μm-pore size filter supports at a densitiy of 2 × 10^5 ^cells/cm^2^. Cells were cultured for 6 days with medium changes every other day until confluence was reached. Then, the cells were infected with NiV at a m.o.i. of 0.5 or left mock-infected. At 6 h or 24 h p.i., horseradish peroxidase (HRP, Sigma) was added to the upper chambers at a final concentration of 5 μg/ml. At different time points after HRP addition (5 min to 2 h), aliquots of 100 μl of medium in the lower chamber were collected, and HRP activity was determined colorimetrically by adsorbance at 470 nm to detect the *O*-phenylenediamine (OPD) reaction product after incubating 20 μl of each sample with 150 μl substrate buffer composed of 0.1 M KH_2_PO_4 _buffer with 0.05 M acidic acid at pH 5 and freshly added 0.012% H_2_O_2 _and OPD (400 μg/ml). Because the initial passage of molecules proceeds linearly in time, the flux of peroxidase was calculated from the initial hour of passage. The mean HRP concentration in the lower chamber medium was normalized to the HRP concentration in the mock-infected control wells, and the results were graphed as means of 3 experiments.

### Surface immunofluorescence analysis

PBMEC and PAEC were grown on fribronectin-coated 0.4 μm-pore size filter supports and infected with NiV. At 24 h p.i., NiV-infected cells were fixed with 4% paraformaldehyde (PFA) in DMEM for 48 h and then incubated from both sides with a polyclonal antiserum from infected guinea pigs (gp4; kindly provided by Heinz Feldmann) or with rabbit monoclonal antibodies directed against the NiV F or the NiV G protein (mab 92 or mab26, respectively; kindly provided by Benhur Lee) for 2 h at 4°C. The primary antibodies were detected using AlexaFluor 568-conjugated secondary antibodies (Invitrogen) for 1.5 h at 4°C. To visualize cell junctions, cells were permeabilized for 10 min with 0,1% Triton in PBS^++ ^and stained with a monoclonal antibody against VE-cadherin (Santa Cruz Biotechnology, Inc.) and AlexaFluor 488-conjugated secondary antibodies (Invitrogen). Filters were cut out from their supports, mounted onto microscope slides in Mowiol 4-88 (Calbiochem) and were analyzed using a Zeiss Axiovert200M microscope or with a confocal laser scanning microscope (Zeiss, LSM510). PAEC stably expressing wildtype or mutant F or G proteins were grown on filter supports and incubated with the polyclonal anti-NiV serum gp3 for 2 h at 4°C without prior fixation. Primary antibodies were visualized using AlexaFluor 568-labeled secondary antibodies (Invitrogen) for 1.5 h at 4°C. PAEC stably expressing EB2 proteins were grown on filter supports and incubated with recombinant mouse EphB4/Fc, a soluble EB2 receptor fused to the FC region of human IgG (R&D Systems) for 2 h at 4°C after fixation with 4% PFA for 15 min at 4°C. Primary antibodies were visualized using AlexaFluor 568-labeled secondary antibodies (Invitrogen) for 1.5 h at 4°C. Confocal fluorescence images were recorded using a Zeiss LSM510 microscope.

### Plasmid construction

cDNA fragments spanning the F and the G genes of the NiV genome (GenBankTM accession number AF212302) were cloned into the pczCFG5 vector as described earlier [35]. By using complementary oligonucleotide primers, tyrosine or leucine residues in the cytoplasmic tails of F and G were changed to alanines to generate the mutants F_Y525A_, F_Y542/543A_, G_Y28/29A _and G_L41/42A _([15] Figure 3A).

Stably EB2-expressing PAEC were constructed as described previously [36] and were kindly provided by H. Augustin.

### Stable glycoprotein expression in PAEC

For stable expression of wildtype and mutant F or G proteins, PAEC were transduced with VSV-G-pseudotyped retroviral vectors carrying the NiV glycoprotein genes. Pseudotypes were produced in 293T cells as described by [37,38]. Briefly, 1.2 × 10^6 ^293T cells were cultured for 16 h prior transfection. Then, 5 ug of the pczCFG-F or -G expression plasmids, 5 μg of the MLV gag-pol encoding pHIT60 plasmid, and 5 μg of the pczCFG-VSV-G plasmid (both kindly provided by J. Schneider-Schaulies) were transfected into the 293T cells by using polyethylenimine [39]. The transfection mixture was replaced by fresh medium after 7 h. At 24 h after transfection, cells were incubated with sodium butyrate for 5 h to induce the CMV promoter of the pczCFG-VSV-G plasmid to increase pseudotype production. Cell supernatants were harvested 48 and 72 h after transfection, filtered through a 0.45 μm pore-size filter (Millipore). Then, 1 ml was directly used for transduction of 1 × 10^6 ^PAEC. To enhance pseudotype binding to the cells, polybrene was added at a concentration of 8 μg/ml. After transduction for 5-16 h, cells were washed and selected for the pczCFG5-encoded zeocin resistance by addition of 0,5 mg of zeocin (InvivoGen) per ml medium. Selected cell clones were screened for stable expression of wildtype and mutant F or G proteins by immunofluorescence analysis.

#### Selective surface biotinylation and immunoprecipitation

PAEC stably expressing either F or G proteins were grown on filter supports. 7 d after seeding, selective surface biotinylation was performed as described recently [40]. Briefly, cells were incubated twice for 20 min at 4°C with 2 mg/ml sulfo-N-hydroxysuccinimidobiotin (S-NHS-biotin; Pierce) at either the apical or the basolateral surfaces. After biotinylation, cells were washed with cold PBS containing 0.1 M glycine and cells were lysed in 0.5 ml of radioimmunoprecipitation assay buffer (1% Triton X-100, 1% sodium deoxycholate, 0.1% sodium dodecyl sulphate [SDS], 0.15 M NaCl, 10 mM EDTA, 10 mM iodoacetamide, 1 mM phenylmethylsulfonyl fluoride, 50 units/ml aprotinin, and 20 mM Tris-HCl, pH 8.5). After centrifugation for 45 min at 19,000 g, supernatants were immunoprecipitated using the NiV-specific antiserum gp3 and 40 μl of a suspension of protein A-Sepharose CL-4B (Sigma). Precipitates were washed and finally suspended in reducing (G protein) or non-reducing (F protein) sample buffer for SDS-polyacrylamide gel electrophoresis (PAGE). Following separation on a 10% gel, proteins were transferred onto nitrocellulose, and biotinylated proteins were detected with streptavidin-biotinylated horseradish peroxidase complex (Amersham Pharmacia Biotech) and enhanced chemiluminescence (Thermo Scientific).

## Competing interests

The authors declare that they have no competing interests.

## Authors' contributions

SE carried out all experiments and helped to draft the manuscript. AM designed the study, helped with the analysis and the interpretation of the data and drafted the manuscript. All authors read and approved the final manuscript.
